# Growth evaluation of several types of energy crops from tropical shrubs species

**DOI:** 10.12688/f1000research.18063.1

**Published:** 2019-03-25

**Authors:** Dwi Susanto, Auliana Auliana, Rudianto Amirta

**Affiliations:** 1 Physiology and Plant Development Laboratory, Department of Biology, Faculty of Mathematic and Natural Sciences, Mulawarman University, Samarinda, East Kalimantan, 75123, Indonesia; 2 Forest Product Technology Laboratory, Faculty of Forestry, Mulawarman University, Samarinda, East Kalimantan, 75123, Indonesia

**Keywords:** Pioneer species, Tropical shrubs, Energy crops

## Abstract

**Background: **Few species of tropical shrubs potentially produce biomass to replace fossil fuels for heat production and electricity. The aims of this study were to determine the growth and nutrient status of leaves of several types of energy crops from tropical shrub species with NPK fertilizer application.

**Methods: **Randomized block design was used with ten replications of four levels of fertilizer treatment: T0 = 40 g, T1 = 80 g, T2 = 120 g and T4 = 160 g per plant.

**Results: **The results indicated that fertilization increased plant growth and the quantity of nutrients in leaves. The plants accumulated a lot of potassium, followed by nitrogen and phosphorus. The species of tropical shrubs with the best growth were *Vernonia amygdalina*, *Calliandra calothyrsus and Gliricidia sepium, *which are all potentially cultivated as sustainable energy crops.

**Conclusions: **Serious attention must be paid to the availability of soil nutrients in order to sustain the cultivation of these plants.

## Introduction

The Indonesian government has issued a national energy policy to encourage the development of alternative energy. This policy targets the replacement of diesel and gasoline with biodiesel and bio-ethanol by 5% in Indonesia by 2025. The Government commissioned the Ministry of Forestry to play an active role in the development of biomass production. The use of raw material biomass to replace fossil fuels for heat and electricity production is prioritized, including permits for the utilization of plantations in the form of unproductive areas and permits for natural forest utilization ^1, 2^.

Different types of forest plants and lignocellulose weeds have the potential to be developed as biomass feedstock for electricity production. Woody shrub species, such as *Vernonia amygdalina* Delile, *Piper aduncum* L., *Gliricidia sepium* (Jacq.) Kunth ex Walp., *Calliandra calothyrsus* Meissner., *Bridelia tomentosa* Blume, *Vitex pinnata* L., *Vernonia arborea* Buch.-Ham. and *Bauhinia purpurea* var. corneri de Wit., are suitable for use as sustainable raw materials for electrical energy ^3^. This type of bush plant is a pioneer species that can easily be grown in secondary forests and open lands that have formerly been cultivated and logged. Energy crops are defined as cultivated plants that are developed and grown specifically for fuel and are rapidly growing, resistant to pests and droughts and can quickly be harvested, so they could have competitive prices if used as fuel ^4^.

Wood is a renewable resource as it is sustainable and its supply will always be available. Meanwhile, cellulotic bio-fuel is produced from non-food (feed) stocks that play a critical role in reducing dependence on oil imports ^5^. Woody biomass is produced, among others, by establishing plantations of energy crops. Coppice is a tree process regenerated with new shoots from the stumps that have been harvested and the Short Rotation Coppice (SRC) from hardwoods is more promising in generating biomass for bio-energy because it consists of fast-growing tree species and high-yield planted varieties (5000-15000 stems per ha), harvested in a two to six year rotation cycle.

This study focused on the growth and nutrient status of leaves of several types of energy crops from tropical shrub species with NPK fertilizer application, as a first step in the preparation of ready-to-plant if they are cultivated in the future as raw materials for biomass for renewable electricity.

## Methods

### Site and time

This study was conducted from January to September 2018 in a secondary forest located at Suka Damai Village, Muara Badak Sub-district, Kutai Kartanegara District, East Kalimantan province, Indonesia (00°17’ to 18°2” S latitude and 117°14’ to 14°39.5” E longitude). The wet season varies from 9 to 12 months and the dry season varies from 0 to 3 months. The average monthly temperature is 27.5 ^°^C and average air humidity is 82% ^6^.

### Plant materials

Seedling plants ( *Vernonia amygdalina, Piper aduncum, Gliricidia sepium, Symplocos fasciculata, Vitex pinnata, Bauhinia purpurea, Melastoma malabathricum,* and *Calliandra calothyrsus)* were taken from the Mulawarman University Botanical Garden. Seedlings are included in polyethylene bags and maintained for 3 months. Those with uniform height are selected before being planted in the research plot.

### Experimental design

**Figure 1.**
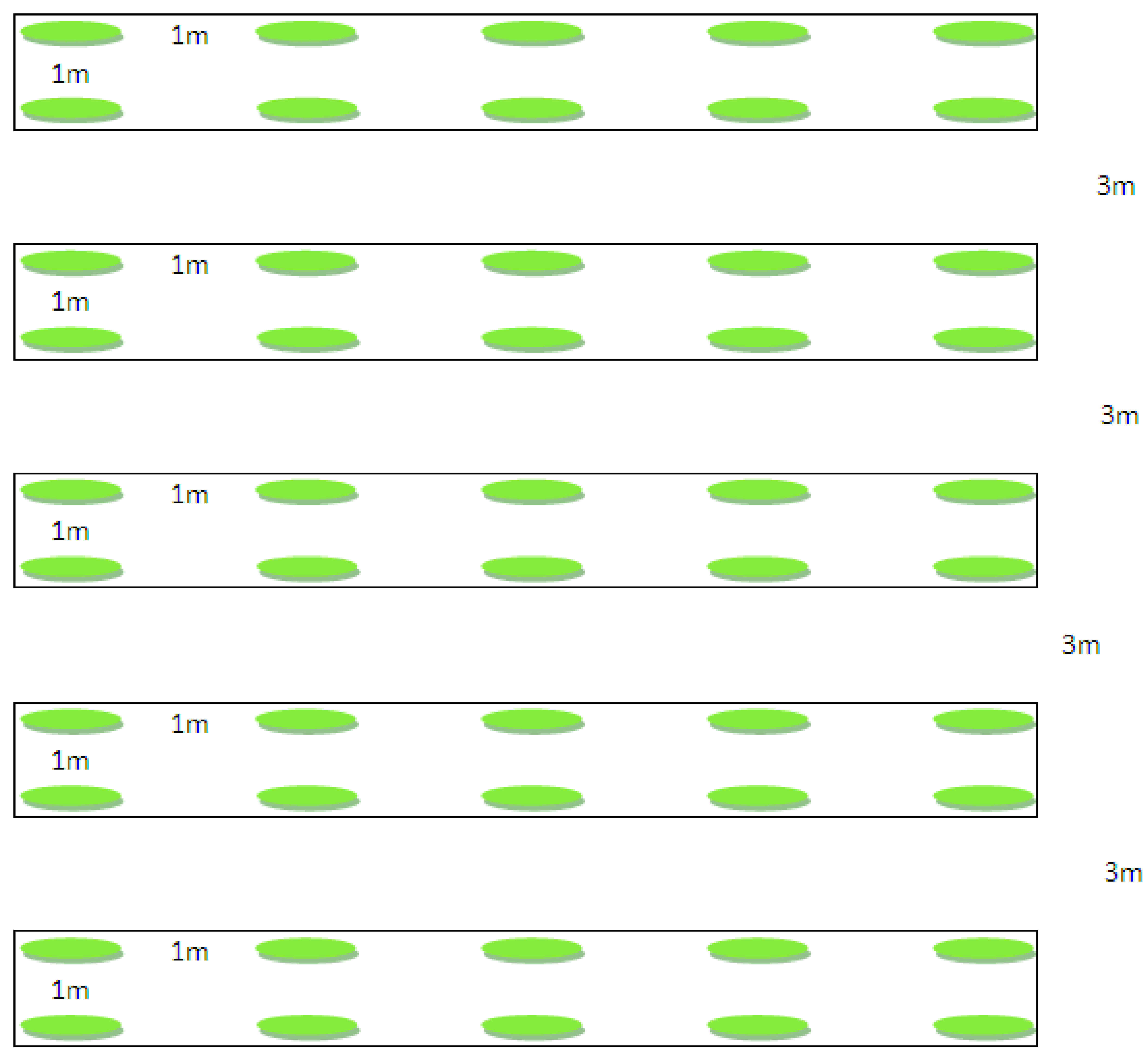
Seedling planting diagrams for each treatment.

Complete randomized block design (CRBD) was used as the experimental design. There were 8 plant species and their growth responses were evaluated based on 5 treatments using NPK fertilizer (commercial by YARA International ASA, Oslo, Norway; 16% N, 16% P _2_O _5_, 16% K _2_O _5_, 1.5% MgO, and 5% CaO). Five groups in five plots (each plant species): T0: with no fertilizer as control group (0 g/plant); T1: supplemented with NPK 40 g/plant; T2: supplemented with NPK 80 g/plant; T3: supplemented with NPK 120 g/plant; T4: supplemented with NPK 160 g/plant. The fertilizer treatments were conducted 2 weeks after sowing. There were 10 sample plants (as replication) for each treatment, a total of 50 plants for each species (divided into 5 experimental plots). Therefore, in total, there were 40 experimental plots, each plant was separated 1 m in length within each plot while it was separated 3 m in length between each plot ( Figure 1).

### Measured parameters

All data were recorded after 5 months of planting. Stem height was measured by retractable tape measure (Shiro, Japan), basal stem diameter was measured by Vernier digital caliper (Mitutoyo, Japan), and the number of leaves and branches of each plant were counted.

All 10 replications of each treatment were pooled into one composited plant sample. Plant materials were analyzed at Forest Soil Science Laboratory, Faculty of Forestry, Mulawarman University to determine the total nitrogen, phosphorus, potassium, calcium and magnesium concentration. Total nitrogen was estimated using the Kjeldahl method ^7^. Briefly, leaves were extracted using the wet destruction method using concentrated H _2_SO _4 _(MERCK, Germany). The extract was distilled and added to 20 ml of 0.05 N NaOH (MERCK, Germany).

To measure the elements of P, K, Ca and Mg, the plant materials were extracted using high pressure digestion method at a temperature of 180 ^0 ^C for 10 hours with HNO _3 _65% (MERCK Milipore, Germany) as a reductant. The calorimetric technique used nitric acid-molybdate-vanadate (MERCK, Germany) as a coloring agent and it was determined by spectrophotometer (GENESYS™20 Visible Spectrophotometer, Thermo Scientific™, Thermo Electron North America LLC, 14-385-445) at a wavelength 470 nm. Meanwhile potassium, calcium and magnesium concentration were measured by Atomic Absorption Spectrophotometer (Trace 1800, Aurora Biomed, Canada) at wavelengths of 766.5, 489.5 and 245.2 nm, respectively ^5^.

### Data analysis

The plant growth data were expressed as mean ± standard error. The data were subjected to ANOVA, followed by Duncan’s Multiple Range Test (DMRT) to evaluated significant differences among the groups of treatment. All analysis was done using SPSS 22 (SPSS Inc. USA) and all significant tests were set at p≤0.05. The data of leaves’ nutrient concentration were analyzed descriptively.

## Results

The first part was the result of planting several types of tropical shrubs, including height growth, stem diameter, number of branches and leaves, while second part included the results of the analysis of nutrient content that was accumulated on the leaves of several types of tropical shrubs, namely nitrogen, phosphorus, potassium, calcium and magnesium.

Based on Table 1, the best stem height was measured from 67.00 cm to 320.50 cm in *V. pinnata* and *V.amygdalina,* respectively, where both results were observed with T4 treatment. The best stem diameter ranged from 0.68 cm to 1.60 cm in *B. purpurea* and *P. aduncum,* respectively. The *B. purpurea* result was observed in T4 treatment, whereas the *P. aduncum* result was in T1 treatment. The highest leaf number ranged from 34.10 in *B. purpurea* to 271.50 in *V.amygdalina*. The first result from *B. purpurea* was observed in T1 treatment while the second from *V. amygdalina* was in T4 treatment. The highest branch number varied from 2.90 in *B. purpurea* to 12.00 in *V. amygdalina*. Similar to the leaves, *B. purpurea* result was observed in T1 treatment while *V. amygdalina* was different, because it was obtained from only T2 treatment. Other results on several plant species also show highest numbers however they are not significant within each species.

**Table 1.** Fertilization effect on the average number of plant height, stem diameter, and leaf and branch number. T0 = 0 g (control), T1 = 40 g, T2 = 80 g, T3 = 120 g and T4 = 160 g of NPK. Number followed by the same letter in the same column show no significant difference in the DMRT test ( *p=0.05*) after analysis by ANOVA.

Plant species	Fertilizer treatment	Stem height	Stem diameter	Leaf number	Branch number
*Vernonia amygdalina*	T0	197.90 ^a^ ± 49.99	1.3020 ^a^ ± 0.66	103.30 ^a^ ± 38.16	6.50 ^a^ ± 1.43
	T1	302.10 ^bc^± 63.39	1.3640 ^a^ ± 0.56	185.70 ^b^ ± 55.00	10.80 ^b^ ± 2.53
	T2	283.60 ^bc^ ± 59.95	1.2710 ^a^ ± 0.38	230.10 ^bc^ ± 63.71	12.00 ^b^ ± 4.03
	T3	260.20 ^b^ ± 55.61	1.0600 ^a^ ± 0.26	194.90 ^b^ ± 51.68	10.90 ^b^ ± 4.23
	T4	320.50 ^c^ ± 51.15	1.3480 ^a^ ± 0.36	271.50 ^c^ ± 53.09	10.80 ^b^ ± 4.76
*Piper aduncum*	T0	61.60 ^a^ ± 17.12	0.97 ^a^ ± 0.41	44.10 ^a^ ± 17.67	6.80 ^a^ ± 2.62
	T1	98.20 ^bc^ ± 31.52	1.60 ^b^± 0.66	89.80 ^b^± 70.00	11.00 ^b^± 4.08
	T2	91.80 ^bc^ ± 17.73	1.24 ^ab^ ± 0.40	59.30 ^ab^ ± 47.45	6.70 ^a^ ± 2.98
	T3	80.60 ^ab^ ± 17.46	0.93 ^a^ ± 0.22	33.90 ^a^ ± 13.08	5.00 ^a^ ± 2.31
	T4	107.40 ^c^ ± 36.69	1.33 ^ab^ ± 0.48	47.50 ^a^ ± 30.10	5.30 ^a^ ± 2.45
*Vitex pinnata*	T0	35.90 ^a^ ± 14.40	0.44 ^a^ ± 0.13	20.30 ^a^ ± 9.55	1.40 ^a^ ± 1.35
	T1	54.20 ^ab^ ± 12.95	0.58 ^a^ ± 0.15	19.00 ^a^ ± 7.99	1.70 ^a^± 0.68
	T2	55.50 ^ab^ ± 16.47	0.53 ^a^ ± 0.11	17.90 ^a^ ± 6.57	1.30 ^a^± 0.48
	T3	54.20 ^ab^ ± 25.07	0.56 ^a^ ± 0.16	15.00 ^a^ ± 4.81	1.40 ^a^± 0.70
	T4	67.00 ^b^ ± 46.01	0.56 ^a^ ± 0.21	16.50 ^a^ ± 7.74	1.20 ^a^± 0.79
*Calliandra calothyrsus*	T0	96.60 ^a^ ± 60.24	0.69 ^a^ ± 0.37	13.70 ^a^ ± 7.85	2.00 ^a^ ± 1.41
	T1	142.80 ^ab^ ± 70.28	0.96 ^ab^ ± 0.37	23.50 ^ab^ ± 13.97	1.50 ^a^± 0.97
	T2	102.50 ^a^ ± 46.97	0.71 ^a^ ± 0.25	20.70 ^ab^± 12.69	1.10 ^a^± 0.57
	T3	163.70 ^b^ ± 53.25	1.10 ^b^± 0.40	30.50 ^ab^ ± 26.86	1.90 ^a^± 1.66
	T4	192.10 ^b^ ± 63.24	1.00 ^ab^ ± 0.34	38.40 ^b^± 24.56	1.60 ^a^± 0.84
*Melastoma malabathricum*	T0	75.70 ^a^ ± 12.54	0.73 ^a^ ± 0.19	76.00 ^a^ ± 36.62	7.30 ^ab^ ± 4.24
	T1	127.10 ^b^ ± 57.89	0.97 ^b^ ± 0.27	129.60 ^b^ ± 78.96	8.80 ^b^ ± 3.85
	T2	99.30 ^ab^ ± 20.04	0.85 ^ab^ ± 0.24	95.00 ^ab^ ± 27.38	6.10 ^ab^± 2.69
	T3	89.40 ^a^ ± 32.85	0.68 ^a^ ± 0.28	67.30 ^a^ ± 42.36	4.60 ^a^± 1.51
	T4	83.60 ^a^ ± 26.65	0.71 ^a^ ± 0.23	66.20 ^a^ ± 40.86	5.40 ^a^± 4.17
*Symplocos fasciculata*	T0	39.30 ^a^ ± 10.82	1.06 ^a^ ± 0.29	29.20 ^a^ ± 14.22	3.40 ^a^ ± 1.35
	T1	51.60 ^b^ ± 17.53	1.02 ^a^ ± 0.38	39.50 ^a^ ± 10.82	3.40 ^a^± 1.08
	T2	65.40 ^c^ ± 13.18	1.21 ^a^ ± 0.30	58.50 ^b^ ± 19.90	5.00 ^a^± 2.21
	T3	67.60 ^c^ ± 14.74	1.19 ^a^ ± 0.34	45.90 ^ab^ ± 22.16	3.30 ^a^± 2.21
	T4	77.20 ^c^ ± 7.41	1.05 ^a^ ± 0.24	60.50 ^b^ ± 25.91	3.50 ^a^± 1.58
*Bauhinia purpurea*	T0	44.40 ^a^ ± 16.47	0.45 ^a^ ± 0.13	15.90 ^a^ ± 10.81	2.10 ^ab^ ± 1.10
	T1	79.50 ^b^ ± 40.11	0.63 ^ab^ ± 0.30	34.10 ^b^± 23.28	2.90 ^b^± 2.69
	T2	66.60 ^ab^ ± 34.31	0.63 ^ab^ ± 0.17	23.50 ^ab^ ± 8.67	1.30 ^a^± 0.82
	T3	46.10 ^a^ ± 15.47	0.49 ^ab^ ± 0.19	22.50 ^ab^ ± 8.00	1.50 ^ab^± 0.85
	T4	69.50 ^ab^ ± 30.58	0.68 ^b^± 0.27	20.10 ^a^ ± 13.92	1.10 ^a^± 1.20
*Gliricidia sepium*	T0	80.00 ^a^ ± 11.92	1.05 ^a^ ± 0.39	16.75 ^a^ ± 5.56	3.50 ^a^ ± 1.92
	T1	162.00 ^b^ ± 44.53	1.37 ^a^ ± 0.54	50.67 ^b^ ± 28.57	4.67 ^a^± 1.53
	T2	179.25 ^b^ ± 50.61	1.67 ^a^ ± 0.46	62.00 ^b^ ± 21.91	4.75 ^a^± 2.22
	T3	170.33 ^b^ ± 52.65	1.65 ^a^ ± 0.31	47.00 ^b^ ± 19.47	4.67 ^a^± 0.58
	T4	192.50 ^b^ ± 46.88	1.64 ^a^ ± 0.34	59.25 ^b^ ± 5.85	4.75 ^a^± 1.26

**Figure 2.**
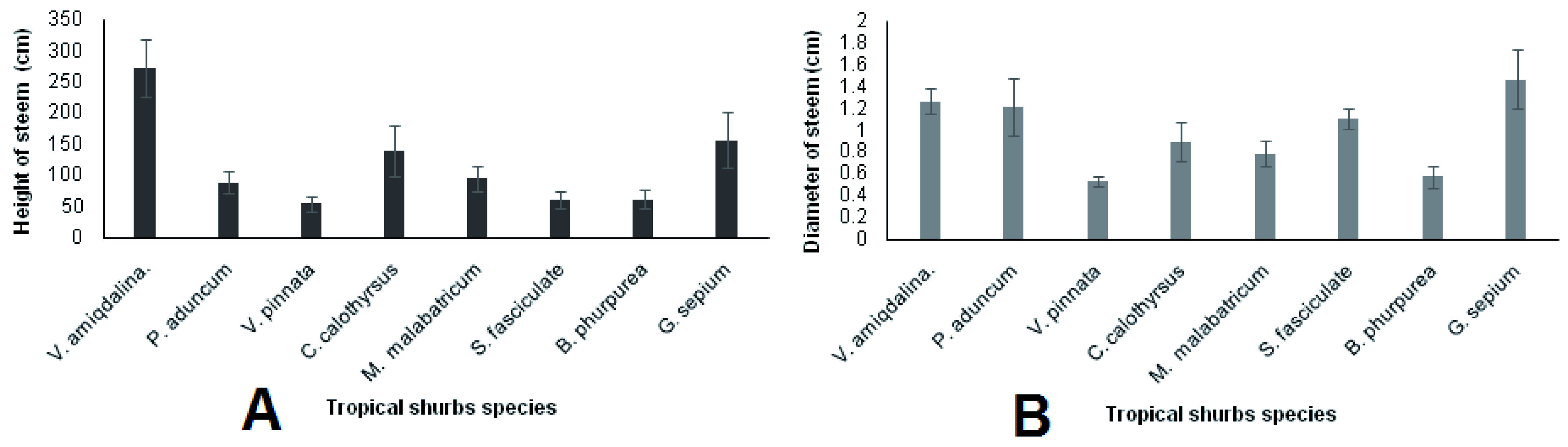
( **A**) Height and ( **B**) diameter comparison of tropical shrub stems 5 months after planting measured from all fertilizer applications.

The results show that fertilization treatment affects the growth of superior energy tropical shrub plants. In plants *V. amygdalina, P. aduncum, V. pinnata, C. callothyrsus, S. fasciculata* and *G. sepium*, the best growth was found in T4 treatment, whereas in *M. malabathricum* and *B. purpurea*, the best growth was obtained by T1 treatment. This indicates that the response of plants varies according to the fertilization treatment. Stem height and stem diameter of the various plants after 5 months of planting are presented in Figure 2.

According to Figure 2, the highest stem height was observed in *V. amygdalina* while the lowest was in *V. pinnata, S. fasciculata* and *B. purpurea.* Moreover, the large stem diameter was measured in *P. aduncum,* while the lowest was in *V. pinnata* and *B. purpurea*.

**Figure 3.**
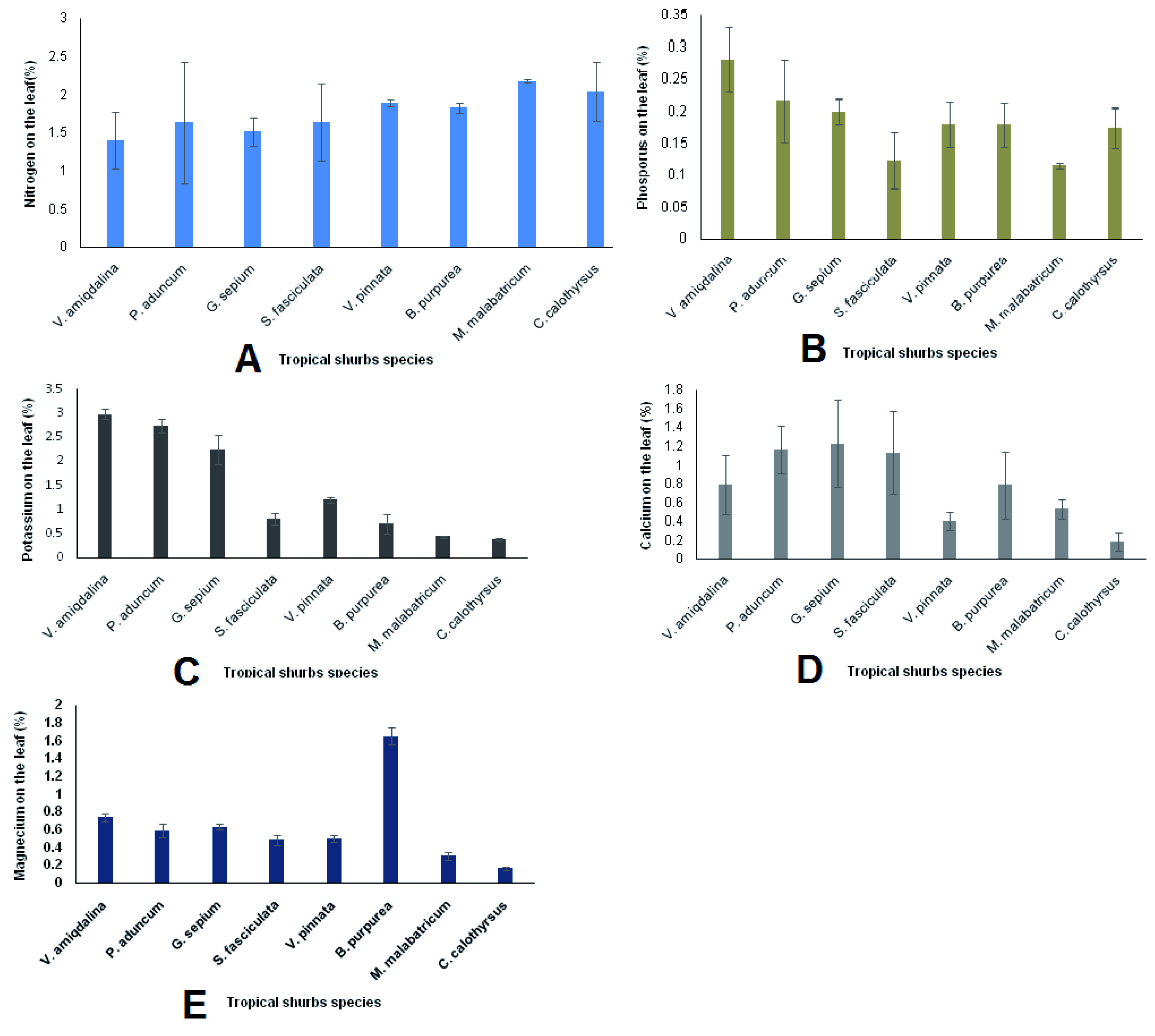
Nutrient accumulation in leaves of tropical shrubs 5 months after planting measured from all fertilizer applications ( **A**. Nitrogen, **B**. Phosphorus, **C**. Potassium, **D**. Calcium, and E. Magnesium).

The accumulation of nutrients in the leaves of the tropical shrub plants varied widely. The highest nitrogenous nutrients accumulated in the leaves of *M. malabatricum, C. calothyrsus* and *V. pinnata,* while the highest phosphorus and potassium nutrients accumulated in the leaves of *V. amygdalina, P. aduncum* and *G. sepium*. Calcium nutrients accumulated the most in the leaves of *P. aduncum, G. sepium* and *S. fasciculata*. On the other hand, the highest accumulation of magnesium nutrients occurred in the leaves of *B. purpurea, V. amiqdalina* and *G. sepium* ( Figure 3). Full data of the effect of fertilizer applications on tropical shrubs growth (height, diameter, and leaf and branch number) and leaves’ nutrient concentration (N, P, K, Ca and Mg) are available ^8^.

## Discussion

In this study, the growth and accumulation of nutrients in the leaves of tropical shrub plants varied greatly. The tropical shrubs that grew the best were *V. amygdalina, G. sepium* and *C. calothyrsus,* which accumulated mostly phosphorus and potassium nutrients in the leaves, while nitrogen, calcium and magnesium were least accumulated. These plants responded to NPK fertilizer up to 160 g per plant, resulting in best growth and high production of biomass, showing their suitable as raw material for biomass to create electricity. A previous study has shown that *V. amygdalina* provides 2.25 MWh, *G. sepium* 2.08 MWh and *C. calothyrsus* 2.01 MWh per ton of dry biomass ^3^. As reported by Susanto and Amirta, fast-growing pioneer species such as *M. gigantea* absorbs the most potassium reaching 35% in every ton of plant biomass ^6, 9^. For *V. amygdalina*, growth parameters are positively correlated to rainfall, relative humidity and cloud cover ^10^. On the other hand, *G. sepium* can be harvested at a residual height of 70 cm, with better agronomic characteristics and chemical composition occurring in the fall ^11^. *C. calothyrsus* also has good growth in planting plots in previous research ^12^, and it was previously reported that mycorrhizae such as *Glomus* sp *.* and *Acaulospora* sp *.* have signiﬁcant influence on its height. In Colombia, planted fallows using *C. calothyrsus* have an additional benefit of producing large quantities of wood for household use ^13^. Based on the growth and nutrient analysis in the present study, we believe that these tree plants species can be developed widely to support a sustainable supply of biomass feedstock for the green electricity program in Indonesia.

The growth of five plants species, namely *M. malabatricum*, *P. aduncum*, *V. pinnata*, *B. purpurea*, *S. fasciculata* was lower (less than half) than the three plants species discussed earlier ( Table 1). In the present study, several plant species that grew slower actually accumulated more nutrients of nitrogen and phosphorus in the leaves, such as *S. fasciculata, P. aduncum, V. pinnata, B. purpurea* and *M. malabatricum*, *P. aduncum* and *S. fasciculata* also accumulated a large amount of potassium and calcium nutrients in their leaves. Moreover *B. purpurea* accumulated magnesium mostly in its leaves ( Figure 3). For *M. malabathricum,* the availability of phosphorus and aluminum in the rhizosphere increases its growth, it can also adapt to low soil pH ^14^ and absorb heavy metals in contaminated soils ^15^. On the other hand, the mean foliar aluminum concentration in wild plants of *M. malabathricum* had positive correlation with foliar calcium, total nitrogen, calcium and magnesium concentrations within this species ^16^ while *Symplocos* sp. mean foliar aluminum concentrations were detected at 4107 (±1474 mg kg ^-1^) and 4290 (±4025 mg kg ^-1^) for seedlings and saplings, respectively ^17^. *P. aduncum* can be propagated with seeds and shoot cuttings ^18^ and can accumulate large amounts of potassium, as previously reported ^19^; at 23 months, it had accumulated 222 kg N, 50 kg P, 686 kg K, 255 kg Ca, 75 kg Mg, and 24 kg S ha ^− 1^. More than half of the P, K, Ca and Mg nutrients were found in the stem (wood). Its leaf litter is significant and becomes an easily decomposable source of potassium, but *G. sepium’s* leaf litter contains much nitrogen ^18^ while *B. purpurea,* which is a light-demanding tree, only grew 25% in a shady house with full sunlight ^20^. Therefore, each tropical shrubs species in this study varies in accumulating plant nutrients N, P, K, Ca and Mg in their leaves.

Based on this study, it is necessary to pay attention to the fast developing plant type, especially for tropical shrubs that have the potential as raw materials for biomass energy. Many beneficial nutrients also accumulated mostly in the leaves. This accumulation reflects the nutrient requirements of those plants, which will be cultivated as energy raw materials; therefore at the initial stage, the demand for plant fertilizer can be predicted. The carrying capacity of soil nutrients requires serious attention for the sustainment of plant cultivation. These wood shrubs species ( *V. amygdalina, G. sepium* and *C. calothyrsus)* were also able to re-grow naturally by generation more than single shoots on their coppice trees. The scheme of Short Rotation Coppices (SRC) was an effort to achieve forest energy plantation using fast growing trees and wood shrubs species for aiming for a sustainable cycle ^3^. 

## Conclusion

Of the eight types of tropical shrubs in this study, three species, namely *V. amygdalina, G. sepium* and *C. calothyrsus,* had the best growth and could potentially be developed as Energy Crops. The most accumulated nutrients in the leaves of these three species of plants are phosphorus and potassium.

## Data availability

Open Science Framework: Growth Evaluation of Several Types of Energy Crops from Tropical Shrubs Species, https://doi.org/10.17605/OSF.IO/3G8FH ^8^.

Data are available under the terms of the Creative Commons Zero "No rights reserved" data waiver (CC0 1.0 Public domain dedication).
