# Supplementary material for: Growth evaluation of several types of energy crops from tropical shrubs species
Source: F1000Res. 2019 Mar 25;8:329. [Version 1] doi: 10.12688/f1000research.18063.1 (PMC8994084; doi:10.12688/f1000research.18063.1)
Supplement: Peer review history

# Peer review: Reviewer response for version 1

**DOI:** 10.5256/f1000research.19754.r127967

**Published:** 2022-04-08

**Authors:** Naqib Ullah Khan

**Affiliations:** 1 Department of Plant Breeding and Genetics, Khyber Pakhtunkhwa Agriculture University, Peshawar, Pakistan

I have gone through the article entitled “Growth evaluation of several types of energy crops from tropical shrubs species” and found it fit for indexing after minor revision. It is a good study, paper is well written, however, there are quite a few grammatical mistakes. It would be better if the language and grammar were checked throughout the text.

 Paper can be Approved after the incorporation of all comments given below:

1. The title is good and interesting for readers.
2. Rewrite the appropriate abstract, lots of similar work is done on this study. What is new in your paper?
3. Overall, the introduction is written poorly. It seems to review the literature. The author failed to justify the need for his research by giving logical arguments. He has cited old references with well-established facts.
4. Needs to add some more recent references. I suggested some references below:
  Fitmawati, Desti, Juliantari E, Novela D, Kapli H (2022) ^1^.
  Abduova AA, Kupriyanov OA, Yessengeldi A, Kupriyanov AN, Sataev MI, Moshkalov BM (2022) ^2^.
Krishna T.P.A., S. Antony Ceasar, T. Maharajan, M. Ramakrishnan, V. Duraipandiyan, N.A. Al-Dhabi and S. Ignacimuthu. 2017.
  Haq, I.U., M. Riaz, A. Nawaz, A.U. Rehman, H. Mukhtar and Q.U.A. Syed. 2020 ^3^.
  Asad, W., T. Kiran, F. Saleem, S. Siddiqui and S.A. Rasool. 2021 ^4^.
  Nan, L.L. and Q.E. Guo. 2021 ^5^.
  Bauyrzhan, T., K. Natalya, I. Zarina, K. Andrey, K. Мeruert, A. Кarime and B. Aliya. 2022 ^6^.
5. The author analyzed the nutrient concentration only in the leaves. Did not give about stem and roots? It is recommended to write a few lines. Also write about the biomass of each plant part.
6. The entire discussion section is lacking an in-depth look at what the driving factors for the results were. Most of this section appears to be a literature review of what others have done and does not draw strong arguments to the data presented. This is in direct contradiction with several statements. This section needs a complete rewrite.

Is the work clearly and accurately presented and does it cite the current literature?

Yes

If applicable, is the statistical analysis and its interpretation appropriate?

Yes

Are all the source data underlying the results available to ensure full reproducibility?

Yes

Is the study design appropriate and is the work technically sound?

Yes

Are the conclusions drawn adequately supported by the results?

Yes

Are sufficient details of methods and analysis provided to allow replication by others?

Yes

Reviewer Expertise:

Crop Breeding and Research...

I confirm that I have read this submission and believe that I have an appropriate level of expertise to confirm that it is of an acceptable scientific standard.

## References

[1] : Molecular phylogeny Of Nibung (Oncosperma tigillarium [Jack] Ridl.) inferred from trnL-F intergenic spacer sequences. *SABRAO Journal of Breeding and Genetics* .2022;54(1) : 10.54910/sabrao2022.54.1.16 175-183 10.54910/sabrao2022.54.1.16

[2] : Coenoflora of Spiraeanthus Schrenkianus (Fisch. And C.A. Mey.) Maxim. *SABRAO Journal of Breeding and Genetics* .2022;54(1) : 10.54910/sabrao2022.54.1.17 184-192 10.54910/sabrao2022.54.1.17

[3] : Enzymatic hydrolysis of Saccharum officinarum Lignocellulosic biomass by genetically modified hyperthermophilic cellulases. *Pakistan Journal of Botany* .2020;52(1) : 10.30848/PJB2020-1(26) 10.30848/PJB2020-1(26)

[4] : Co-optimization of B. licheniformis 208 biomass and alpha amylase synthesis using response surface methodology. *Pakistan Journal of Botany* .2021;53(6) : 10.30848/PJB2021-6(33) 10.30848/PJB2021-6(33)

[5] : Distribution of soil seed reserve and its association with aboveground vegetation in salinized soils of arid regions in northwest China. *Pakistan Journal of Botany* .2021;53(2) : 10.30848/PJB2021-2(29) 10.30848/PJB2021-2(29)

[6] : Phytochemical study of Crambe tatarica Sebeok, a promising source of medicinal plant extracts. *Pakistan Journal of Botany* .2022;54(2) : 10.30848/PJB2022-2(38) 10.30848/PJB2022-2(38)

# Peer review: Reviewer response for version 1

**DOI:** 10.5256/f1000research.19754.r46151

**Published:** 2019-10-31

**Authors:** M. Khairil, Y. Nornasuha

**Affiliations:** 1 School of Agriculture Science and Biotechnology, Faculty of Bioresources and Food Industry, Universiti Sultan Zainal Abidin (UniSZA), Besut, Malaysia; 2 Universiti Sultan Zainal Abidin, Besut, Malaysia

- The abstract needs to have a clear result of the metal elements instead of using 'a lot'. 
- Introduction: I would suggest the authors add some description or mechanism of plants producing energy or electricity. This is optional.
- The method is fine.
- Results: the author analyses the nutrient concentration in the leaves. How about the stem and roots? Is there any specific reason to not include the nutrient concentrations in the stem and roots?
- How about the biomass of each plant part?
- The conclusion is fine.

Is the work clearly and accurately presented and does it cite the current literature?

Yes

If applicable, is the statistical analysis and its interpretation appropriate?

Yes

Are all the source data underlying the results available to ensure full reproducibility?

Partly

Is the study design appropriate and is the work technically sound?

Yes

Are the conclusions drawn adequately supported by the results?

Yes

Are sufficient details of methods and analysis provided to allow replication by others?

Partly

Reviewer Expertise:

Ecology, Botany, Biodiversity and Plant Eco-physiology

We confirm that we have read this submission and believe that we have an appropriate level of expertise to confirm that it is of an acceptable scientific standard.
